# Multiple myeloma presenting in association with gastric phytobezoar

**DOI:** 10.1002/ccr3.1104

**Published:** 2017-07-27

**Authors:** Elizabeth S. Appleton, Natasha A. Lee, Alexander C. Ford

**Affiliations:** ^1^ Leeds Gastroenterology Institute St. James's University Hospital Leeds UK; ^2^ Leeds Institute of Biomedical and Clinical Sciences University of Leeds Leeds UK

**Keywords:** Myeloma, phytobezoar, vomiting, weight loss

## Abstract

We present a rare case of a patient with delayed gastric emptying, gastric phytobezoar formation, and osteosclerotic bone lesions as an atypical association with multiple myeloma. Associated gastric features in myeloma, which include diffuse infiltration, gastric plasmacytomas, or delayed gastric emptying, are rare and have a poor prognosis.

## Introduction

Bezoars are foreign bodies, consisting of animal or vegetable material, and can occur anywhere in the gastrointestinal (GI) tract. The stomach is one of the commonest sites for bezoar formation 1, often as a result of delayed gastric emptying, or gastroparesis. Phytobezoars consist of undigested food, vegetable fiber, or seeds. The management of these includes attempted dissolution, using proteolytic enzymes or carbonated beverages, such as coca‐cola 2. If these measures fail, gastric lavage with coca‐cola 2, endoscopic removal 3, or even surgery 4 may be required. We report an interesting case of a patient presenting with a gastric phytobezoar with rare associated features.

## Case History and Examination

**Figure 1:**
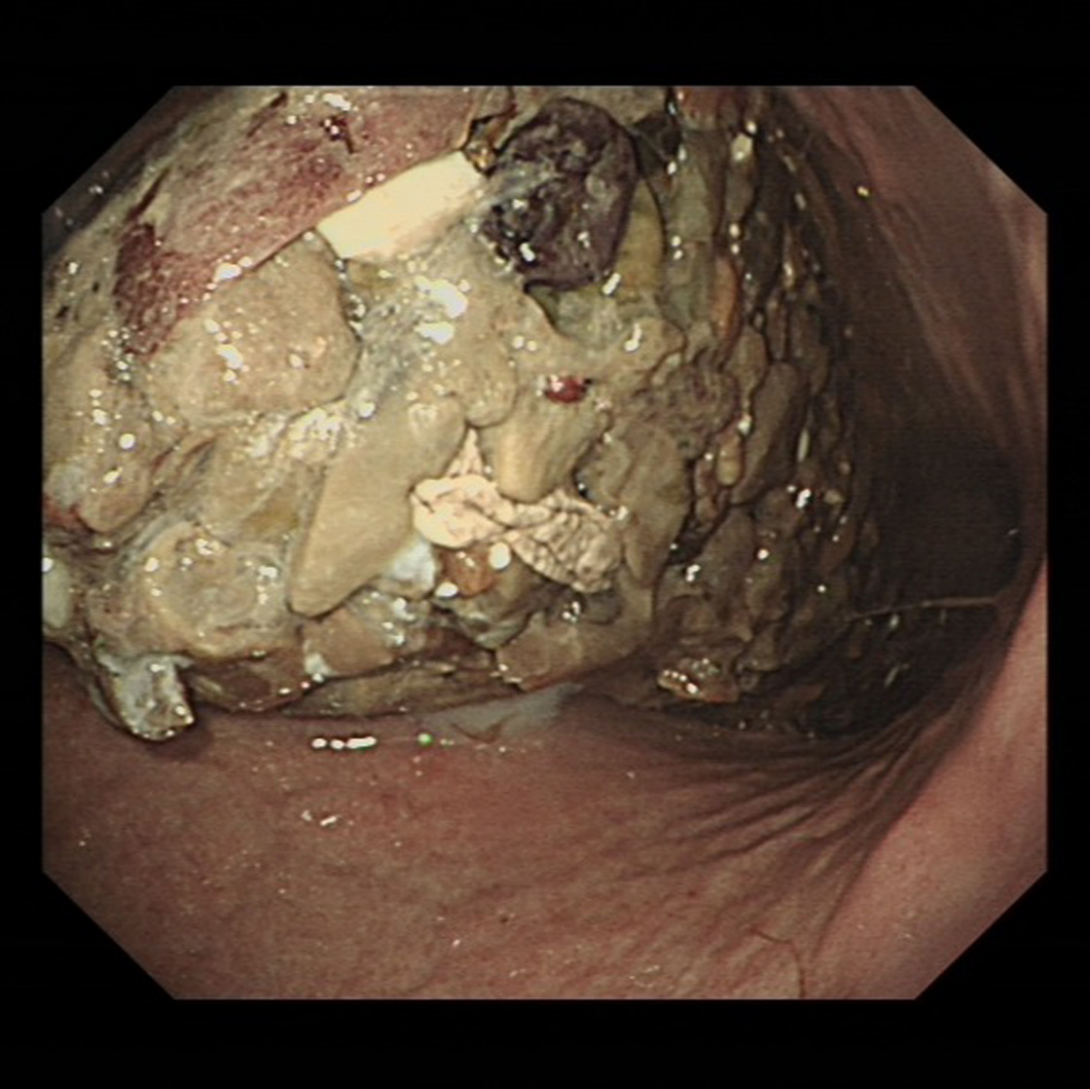
Gastric phytobezoar seen at upper GI endoscopy.

Recently, a 62‐year‐old, previously fit and well, White Caucasian woman presented to our department with a 4‐month history of epigastric pain, vomiting, weight loss of 7 kg, and fatigue. Her index upper GI endoscopy revealed a large gastric phytobezoar (Fig. 1), precluding adequate assessment of the rest of the upper GI tract. Serial upper GI endoscopies, performed as a result of the inability to rule out a structural abnormality, demonstrated persistence of the phytobezoar. Due to continuing weight loss, she was admitted for inpatient investigation, and attempted clearance of the phytobezoar using coca‐cola and pancreatic enzyme supplementation, as described by others 1. There were no relevant findings on clinical examination.

## Investigations and Treatment

Initial laboratory investigations revealed normocytic anemia (hemoglobin 97 g/L [range 115–160 g/L]), low albumin (27 g/L [range 35–50 g/L]), low sodium (126 mmol/L [range 133–146 mmol/L]), and an elevated erythrocyte sedimentation rate (38 mm/h [range 1–15 mm/h]). Further notable results include an elevated urine protein/creatinine ratio of 34 mg/mmol (range 0.1–13 mg/mmol), later 726 mg/mmol, with normal renal function, and paired urine and serum osmolalities consistent with syndrome of inappropriate antidiuretic hormone secretion. Thyroid function tests and serum calcium were normal, although the patient later became hypercalcemic during admission (3.1 mmol/L [range 2.2–2.6 mmol/L]).

**Figure 2:**
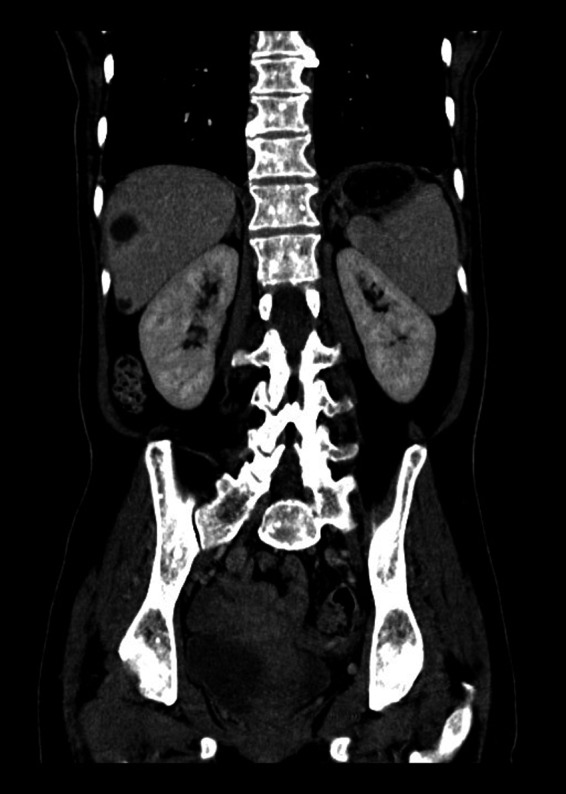
Osteosclerotic lesions seen on CT chest, abdomen, and pelvis.

A chest X‐ray and computed tomography (CT) of head were normal. A CT of the chest, abdomen, and pelvis revealed a grossly distended stomach, with no evidence of structural gastric outlet obstruction, along with diffuse sclerotic bone lesions throughout the spine and pelvis (Fig. 2), thought to be due to benign osteopoikilosis. An isotope bone scan was normal. After a period of enteral feeding via an endoscopically inserted nasojejunal tube, eventual breakdown of the bezoar enabled endoscopic examination of the entire upper GI tract, which was structurally normal. Gastric biopsies were obtained, and these showed mild focal intestinal metaplasia and glandular atrophy only.

Due to persisting hyponatremia, serum immunoglobulins were requested and revealed an elevated IgG of 49.2 g/L (range 6–16 g/L), with low IgM and IgA levels. Serum electrophoresis demonstrated an IgG Lambda paraprotein band of 35 g/L, with Lambda Bence‐Jones protein in the urine, and an elevated β_2_‐microglobulin (5.50 mg/mL [range 1.09–2.53 mg/mL]). The diagnosis of multiple myeloma was confirmed via bone marrow biopsy, which demonstrated 17% plasma cells. Unfortunately, the patient died 1 month after the diagnosis was confirmed, and before appropriate therapy could be instituted.

## Discussion

Multiple myeloma is the second commonest hematological malignancy 5 and is notoriously insidious in its presentation. Largely a disease of older adults 6, 7, the condition often presents with nonspecific symptoms, such as fatigue and weight loss 8. As a disorder primarily of plasma cells, it is a proliferation of malignant B cells within the bone marrow, with deposition of high levels of abnormal immunoglobulin in the blood and urine, leading to osseous manifestations and end‐organ failure. The classic skeletal appearances are of lytic lesions, usually in the pelvis, skull, spine, and long bones, due to malignant deregulation of osteoclastic bone destruction and osteoblast inhibition. However, bone sclerosis, as seen in this patient, is recognized as a primary radiological finding in 3% of patients 9, often associated with myeloma with Lambda chain predominance 10.

Bone disease is evident in at least 80% of myeloma patients at diagnosis. However, the extraskeletal manifestations are wide ranging. Gastric involvement, which may include diffuse infiltration, gastric plasmacytomas, or a clinical picture compatible with delayed gastric emptying, perhaps leading to the phytobezoar seen in this patient with an otherwise structurally normal upper GI tract, is rare and has a poor prognosis 11, 12. In this case, the patient died 1 month later, precluding any formal assessment of gastric emptying time. The association between myeloma and gastroparesis may be due to deposition of amyloid, leading to dysmotility and impaired transit 13. Re‐examination of gastric biopsies, in light of the diagnosis of myeloma, did not reveal any evidence of amyloid deposition, although these contained insufficient submucosa to definitively exclude it.

Osteosclerotic lesions and delayed gastric emptying, with resultant phytobezoar formation, are therefore rarely associated with myeloma, highlighting an atypical presentation of symptoms and clinical findings, and a poor prognosis of these associated gastric features in patients with the condition.

## Authorship

ESA, NAL, and ACF: conceived and drafted the case report. ESA and NAL: collected all data. ESA, NAL, and ACF: drafted the manuscript. All authors contributed to and approved the final draft of the manuscript.

## Conflict of Interest

None declared.
